# Determination of the 3D Atomic Structures of Nanoparticles

**DOI:** 10.1002/smsc.202000045

**Published:** 2020-12-13

**Authors:** Byung Hyo Kim, Junyoung Heo, Jungwon Park

**Affiliations:** ^1^ Department of Fiber Engineering and Organic Materials Soongsil University Seoul 06978 Republic of Korea; ^2^ Center for Nanoparticle Research Institute for Basic Science (IBS) Seoul 08826 Republic of Korea; ^3^ School of Chemical and Biological Engineering Institute of Chemical Process Seoul National University Seoul 08826 Republic of Korea

**Keywords:** 3D electron tomography, 3D reconstruction, atomic coordinate, Brownian one particle reconstruction, liquid-phase transmission electron microscopy, scanning transmission electron microscopy, transmission electron microscopy

## Abstract

The 3D atomic arrangements of materials determine the free energy landscape, thus governing the physical and catalytic properties of those materials. The 3D structures of nanoparticles can deviate from the periodic atomic arrangement of their bulk counterparts due to the dominance of surface dangling bonds, defects, and dislocations. One approach to understand the structure of nanoparticles and their resulting unique properties involves precise probing of the 3D positions of all constituent atoms of individual nanoparticles. The 3D electron tomography and Brownian one particle reconstruction allow investigation of the 3D atomic positions of nanoparticles. Both methods use transmission electron microscopy (TEM) or scanning TEM (STEM) images of nanoparticles with different projection angles and collect their phase information in reciprocal space to reconstruct the 3D structure of the particles. The thus‐reconstructed 3D maps of metal nanoparticles are highly resolved, facilitating the determination of their atomic coordinates. Grain boundary, dislocation, and lattice expansion are observed on the 3D atomic maps. On the basis of the 3D atomic maps, the physical properties of individual nanoparticles can be accurately predicted, enabling purpose‐driven synthesis.

## Graphical abstract

The 3D atomic structures of individual nanoparticles are successfully determined by electron microscopy based methodologies including 3D electron tomography and Brownian one particle reconstruction. This perspective reviews recent development of the methodologies and provides perspectives on the future direction.

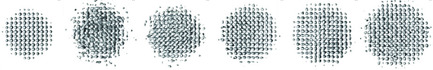

## 1 Introduction

Nanoscience has developed over the last three decades. The development is largely driven by the intriguing properties of nanoparticles that are not observed in their bulk counterparts.^[^ ^1^ ^]^ The newly exhibited properties of nanoparticles are attributable to their small size, high surface‐to‐volume ratio, and quantum effects from the small size.^[^ ^1^, ^2^, ^3^, ^4^, ^5^ ^]^ Superparamagnetism of magnetic nanoparticles, the magnetic state of zero coercivity with high magnetization value, is one of the examples that the small size of nanoparticles matters, because it occurs when the thermal energy exceeds the anisotropy energy, that is proportional to the particle volume.^[^ ^3^, ^4^, ^6^ ^]^ Localized surface plasmon resonance of nanoparticles is originated by the small volume of nanoparticles, and the property is used as sensors,^[^ ^7^, ^8^ ^]^ smart windows,^[^ ^9^ ^]^ and photocatalysts.^[^ ^10^ ^]^ Because of the high surface‐to‐volume ratio, nanoparticles usually show high catalytic effect.^[^ ^11^, ^12^ ^]^ The quantum effect arising at the nanometer scale is responsible for the bandgap tuning of semiconductor nanoparticles.^[^ ^2^ ^]^ It stands to reason that the unique properties of nanoparticles are highly dependent on their 3D atomic structure.

Except few examples,^[^ ^13^ ^]^ nanoparticles synthesized by diverse methods vary in shape, size, and atomic structure despite intensive efforts to achieve homogeneity.^[^ ^14^, ^15^ ^]^ This indicates that the physical and chemical properties of synthesized nanoparticles are determined by the integrated or averaged properties of a heterogeneous ensemble of nanoparticles. Therefore, an in‐depth understanding of the structures of individual nanoparticles is a prerequisite to the ultimate prediction and investigation of the properties of nanoparticle ensembles.

In addition to heterogeneity in overall morphology, individual nanoparticles show unique 3D atomic structures that deviate from their bulk counterparts.^[^ ^16^, ^17^, ^18^ ^]^ A bulk structure is mostly determined by the favored energy state, that is directed by Coulomb potential, bonding energy, and repulsion energy between atoms.^[^ ^19^ ^]^ Crystals, the constituent atoms of which are arranged in an orderly array, are generally energetically stable in bulk materials. Meanwhile, in nanoparticles, surface energy becomes the predominant factor that governs the total energetics of the material.^[^ ^20^ ^]^ As a result, the crystal structure of nanoparticles can differ from the ordered crystal structures of their bulk counterparts.^[^ ^21^, ^22^, ^23^, ^24^, ^25^ ^]^ These structural deviations are also sensitive to the local environment on the surface, such as the specific binding chemistry of ligands that passivate surfaces and interacting molecules in the solution or gas phase.^[^ ^25^, ^26^, ^27^ ^]^ Such change in ligands often leads to an increase or decrease in lattice parameters.^[^ ^27^ ^]^ In addition, the high surface energy of nanoparticles is strongly correlated with the introduction of defects, including vacancies, stacking faults, and dislocations.^[^ ^28^ ^]^

The strong structure‐dependent properties and structural heterogeneity of nanoparticles highlight the importance of understanding the structures of individual particles with 3D atomic resolution. However, the inherent heterogeneity of nanoparticles and their deviation from perfect crystallinity present challenges to understand the 3D structures of nanoparticles using the conventional methods applied to bulk crystalline materials, such as X‐ray diffraction, small angle X‐ray scattering, and Raman spectroscopy.^[^ ^29^, ^30^, ^31^, ^32^ ^]^ Although atom probe tomography (APT) can be used to determine the 3D structure of a tip specimen, spatial resolution of the method is not high enough to assign the atomic positions of constituent atoms.^[^ ^33^, ^34^ ^]^ The 3D structures of nanoparticles have also been obtained by coherent diffractive imaging, but the method relies on a coherent X‐ray beam, which is subjected to technical limitations.^[^ ^35^, ^36^, ^37^ ^]^

**Figure 1:**
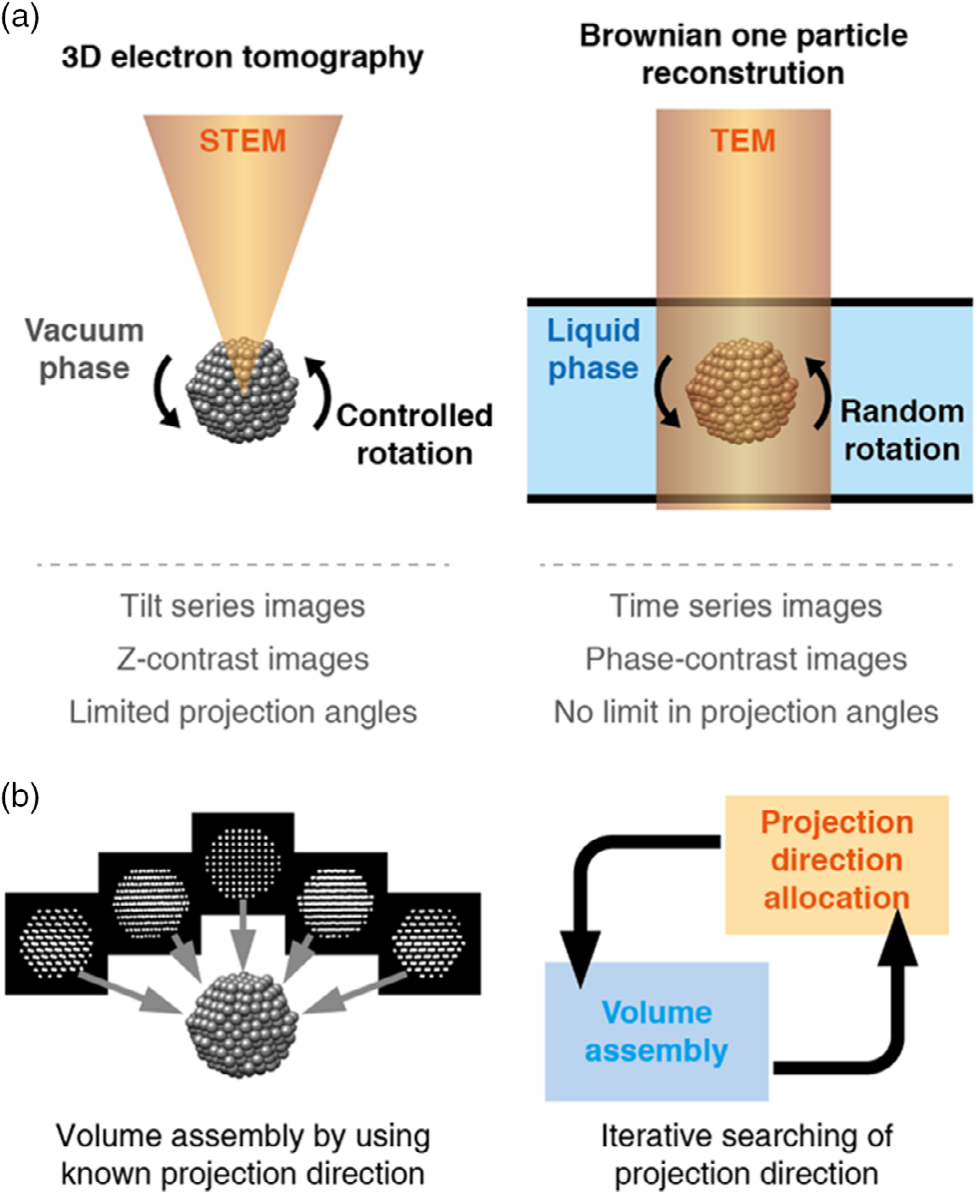
Brief illustration of difference between 3D electron tomography and Brownian one particle reconstruction in a) image acquisition step and b) 3D reconstruction step.

Transmission electron microscopy (TEM) is one of the most widely used methods of analyzing the structure of single nanoparticles because of the directness of the resulting data.^[^ ^38^, ^39^ ^]^ Furthermore, a high‐resolution TEM analysis reveals the atomic positions of nanoparticles projected by an electron beam in 2D.^[^ ^40^ ^]^ TEM‐based analytic methods, including bright‐field TEM, scanning TEM (STEM), electron diffraction, and holography, have reached atomic resolution, but these methods have mainly been applied to 2D mapping of material structures.^[^ ^41^, ^42^ ^]^ Cryo‐electron microscopy (EM) has emerged as an efficient method of reconstructing the 3D structures of biological samples based on imaging specimens fixed in vitrified ice at cryogenic temperatures.^[^ ^43^, ^44^ ^]^ In spite of the remarkable success of cryo‐EM in the field of structural biology—recognized in 2018, when the Nobel Prize in Chemistry was awarded to the developers of the method—the technique is limited to an analysis of the 3D structures of particles that are essentially homogeneous. In heterogeneous structures, the reconstruction process is hampered by the need for intensive classification to sort out the images of non‐identical particles. In cryo‐TEM, the 3D structure of a protein of interest is reconstructed from thousands of TEM images acquired from thousands of individually fixed protein molecules whose projection angles are randomly distributed. Under the assumption that all protein molecules have the same structure, the thousands of TEM images can be reconstructed into one structure. Single‐particle reconstruction based on cryo‐TEM can be used for inorganic nanoparticles from a technical standpoint, but its application is restricted to systems in which the nanoparticle population shares the same crystal structure. For example, homogeneous nanoparticles, such as gold nanoclusters (Au_68_), can be reconstructed by single‐particle reconstruction based on cryo‐TEM.^[^ ^45^ ^]^ Given that most inorganic nanoparticles have heterogeneous structures, TEM images obtained from different individual nanoparticles are independent of one another. Therefore, a method of independently reconstructing 3D structures of different individual nanoparticles is necessary. To obtain the 3D structures of nanoparticles, a significant number of TEM images of a single nanoparticle with different projection angles are required. Two representative methodologies have recently been developed, 3D electron tomography^[^ ^46^ ^]^ and Brownian one particle reconstruction.^[^ ^27^ ^]^ Including these two methods, a few structure analysis methods used for nanoparticles are introduced in **Table** **1**.^[^ ^13^ ^]^ In this perspective, we have major focus on two methods for the 3D structure analysis, 3D electron tomography, and Brownian one particle reconstruction. **Figure** **1** shows the brief scheme and differences of those two methods.

**Table 1:** Comparison between the 3D structure analysis methods

Name of method	Advantages	Disadvantages
Coherent X‐ray diffraction imaging (CXDI)	No Aberration (no lenses used)	Technical limitation to obtain coherent beam
3D Atom probe tomography (APT)	Efficient element assignment (time‐of‐flight‐based discrimination)	Limitation in sample morphology (tip‐shaped sample)
		Sample in high vacuum
Single particle analysis using cryo‐EM	Suitability for both amorphous and crystalline materials	Limitation in sample type (requires sample homogeneity)
3D electron tomography	Individual particle analysis	Missing wedge problem
	Efficient element assignment (Z‐contrast imaging, combined with elemental analysis)	Sample in high vacuum
Brownian one particle reconstruction	Individual particle analysis	Sample degradation by liquid and e‐beam
	Sample in native condition (liquid)	
	No missing wedge	

## 2 3D Electron Tomography

**Figure 2:**
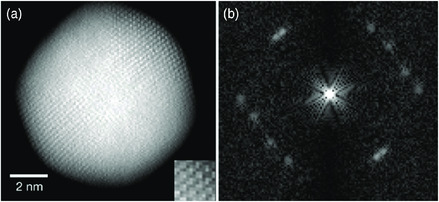
a) 3D volume renderings of a gold nanoparticle and b) its fast Fourier transform patterns along its direction. Reproduced with permission.^[^ ^50^ ^]^ Copyright 2012, Springer Nature.

**Figure 3:**
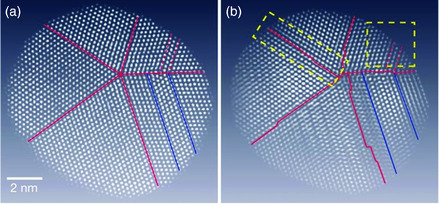
a) STEM image of a platinum nanoparticle with a decahedral shape. The image shows flat grain boundaries. b) Internal atomic layer of the nanoparticle according to the atomic coordinates reconstructed by 3D electron tomography. The sliced atomic map shows stepped grain boundaries. Reproduced with permission.^[^ ^51^ ^]^ Copyright 2013, Springer Nature.

The 3D electron tomography was first developed in 1968 to image frozen biological samples; its use has since been extended to different types of materials, including nanoparticles.^[^ ^47^, ^48^ ^]^ In 3D electron tomography, tens of images of a nanoparticle with different projection angles are obtained with high‐resolution STEM while the particle is manually tilted.^[^ ^46^ ^]^ The 3D nanoparticle maps are reconstructed by combining the images in reciprocal space and inverse Fourier transformation of the result. This process is repeated tens times until the reconstructed 3D density map converges. The spatial resolution of the 3D volume map obtained by 3D tomography is highly dependent on the resolution of the original STEM images and the range of the tilting angles. The resolution of 2D STEM image reaches ≈0.05 nm in attributed to the development of correcting geometrical aberration arising from the electron lenses.^[^ ^49^ ^]^ The nanoparticle images with atomic resolution that have been acquired from aberration‐corrected STEM have been used to produce 3D density maps with improved resolution. Scott et al. obtained the 3D structure of a gold nanoparticle with 0.24 nm resolution, which is sufficient to determine the position of the individual constituent atoms of the nanoparticle.^[^ ^50^ ^]^ The 3D structure shows that the particle has a multiply twinned icosahedral shape (**Figure** **2**). The 3D electron tomography reveals defect structures that are not clearly shown through conventional methods. Chen et al. reported the 3D atomic structure of a multiply twinned decahedral platinum nanoparticle.^[^ ^51^ ^]^ The grain boundary of the twin appears flat on 2D imaging, but the corresponding 3D map reveals that the boundaries are atomic steps rather than flat surfaces (**Figure** **3**). In addition to the atomic step, a stacking fault is seen in the 3D reconstruction. Edge and screw dislocations are also observed in the 3D atomic map. The internal structure of the nanoparticle, represented as a sliced 3D map, shows a zigzag pattern, which is a characteristic feature of screw dislocation. Tian et al. reported the 3D atomic structure of Re‐doped MoS_2_ with a precision down to 4 pm.^[^ ^52^ ^]^ The positions and atomic level details of dopants, vacancies, and ripples of the 2D metal dichalcogenide are presented. Electronic band structures are calculated from experimentally obtained coordinates, enabling to find correlation between structure and electrical properties. In a recent study conducted by Wang et al., the resolution of 3D map from 3D electron tomography reached at 0.053 nm resolution, which is similar to the resolution of 2D STEM images.^[^ ^53^ ^]^ The 3D atomic arrangement of grain boundaries in nanoporous gold is resolved using the 3D electron tomography. Thus, electron tomography now becomes an emerging technique for characterizing diverse functional nanomaterials.^[^ ^54^ ^]^

The spatial resolution of reconstructed 3D density maps can also be improved by maximizing the coverage of the projection angles. Regarding geometric constraints, the tilting angle of a sample is restricted to 79^°^, and the larger angles are referred to as missing wedge angles. Xu et al. overcame the missing wedge angle problem using a needle‐shaped sample.^[^ ^55^ ^]^ The needle‐shaped sample was rotated in a full range from 0° to 180° around the [100] direction with an equal slope increase, and atom‐resolved STEM images with full projection angles were successfully acquired. The tilt series STEM images were reconstructed to 3D structures in reciprocal spaces. The resulting 3D density maps were sufficiently highly resolved to determine the 3D atomic coordinates of the particles. From the 3D atomic coordinates, the displacement field and strain tensors of the individual atoms at the tungsten needle tip were obtained.

**Figure 4:**
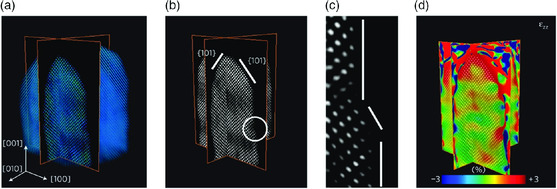
a) 3D reconstruction of a gold nanorod reconstructed by compressed sensing EM. b) Sliced 3D map of the nanorod, showing the {101} surface facets. c) Magnified image of the region encircled in (b). An atomic step with a thickness of two atomic layers is shown on the surface. d) Slice image of *ε*_xx_ strain map revealing tensile strain at the tip of the nanorod. Reproduced with permission.^[^ ^58^ ^]^ Copyright 2012, Springer Nature.

The 3D electron tomography can incorporate additional constraints. Compressed sensing electron tomography can be used to reduce the number of input STEM images. Compressive sensing is a signal processing technique that reconstructs a signal with sparsity by finding solutions to underdetermined linear systems.^[^ ^56^, ^57^ ^]^ The 3D reconstruction using compressed sensing is valid, as only a limited number of voxels contain an atom and most of the voxels are zero‐valued. Only four STEM images were required to reconstruct the 3D structures of gold nanorods (**Figure** **4**).^[^ ^58^ ^]^ A good‐resolution 3D structure was obtained from a few STEM images, allowing observation of surface steps with a thickness of two atomic layers. Using compressed sensing electron tomography, complex‐structured bimetallic nanoparticles, such as Au@Ag core@shell nanorods or decorated heterostructure nanocrystals, can be reconstructed from a few number of STEM images.^[^ ^59^, ^60^ ^]^ Metal chalcogenides can also be reconstructed using compressed sensing electron tomography.^[^ ^61^ ^]^ Various undersampling approaches have been proposed for compressed sensing electron tomography.^[^ ^62^ ^]^ However, finding an appropriate domain for the reconstruction remains challenging.

## 3 Brownian One Particle Reconstruction

Brownian one particle reconstruction is an alternative approach to obtain the 3D structures of inhomogeneous nanoparticles.^[^ ^27^ ^]^ A series of images of nanoparticles with different projection angles in a liquid cell can be acquired over time, because nanoparticles intrinsically rotate in random directions in solution. Liquid cells are fabricated by sandwiching nanoparticle‐containing dispersions between two sheets of graphene; only a small fraction of electrons is wasted by background scattering.^[^ ^63^ ^]^ Increasing the scanning rate of the detector improves the spatial resolution of the TEM images of quickly rotating nanoparticles by reducing rotation‐averaged blurring. For example, use of a direct electron detector enables acquisition of high‐resolution TEM images with a millisecond frame rate, which successfully reduces blurring of lattice fringes of rotating nanoparticles. In addition, the direct electron detector shows a higher signal‐to‐noise ratio compared with a conventional charge‐coupled device detector, improving the resolution of reconstructed 3D maps. Park et al. obtained the 3D maps of two platinum nanoparticles rotating in a graphene liquid cell using the same reconstruction algorithm as in cryo‐EM.^[^ ^64^ ^]^ A stochastic hill climbing method was used to ensure that the reconstructed map did not fall into a local optimum 3D structure, and instead obtained a global optimum structure.^[^ ^65^ ^]^ The resulting near‐atomic 3D maps show the non‐symmetric structures of synthesized metal nanoparticles in colloidal suspension.

Assignment of the 3D atomic coordinates of small nanoparticles in a solvent has been achieved using a large number of high‐resolution TEM images acquired with an enhanced scanning rate and developing an optimized reconstruction algorithm designed for inorganic nanoparticles in a liquid cell. Kim et al. obtained the 3D density maps of eight platinum nanoparticles from the same batch, created via colloidal synthesis.^[^ ^27^ ^]^ The nanoparticles were non‐uniform, ranging in size from 2.2 to 2.9 nm. Six of the nanoparticles showed face‐centered cubic (fcc) structures with slight deformation, and the others deviated from a perfect fcc structure. The 3D atomic positions were assigned with a precision of 19 pm, allowing direct investigation of local atomic structures.

**Figure 5:**
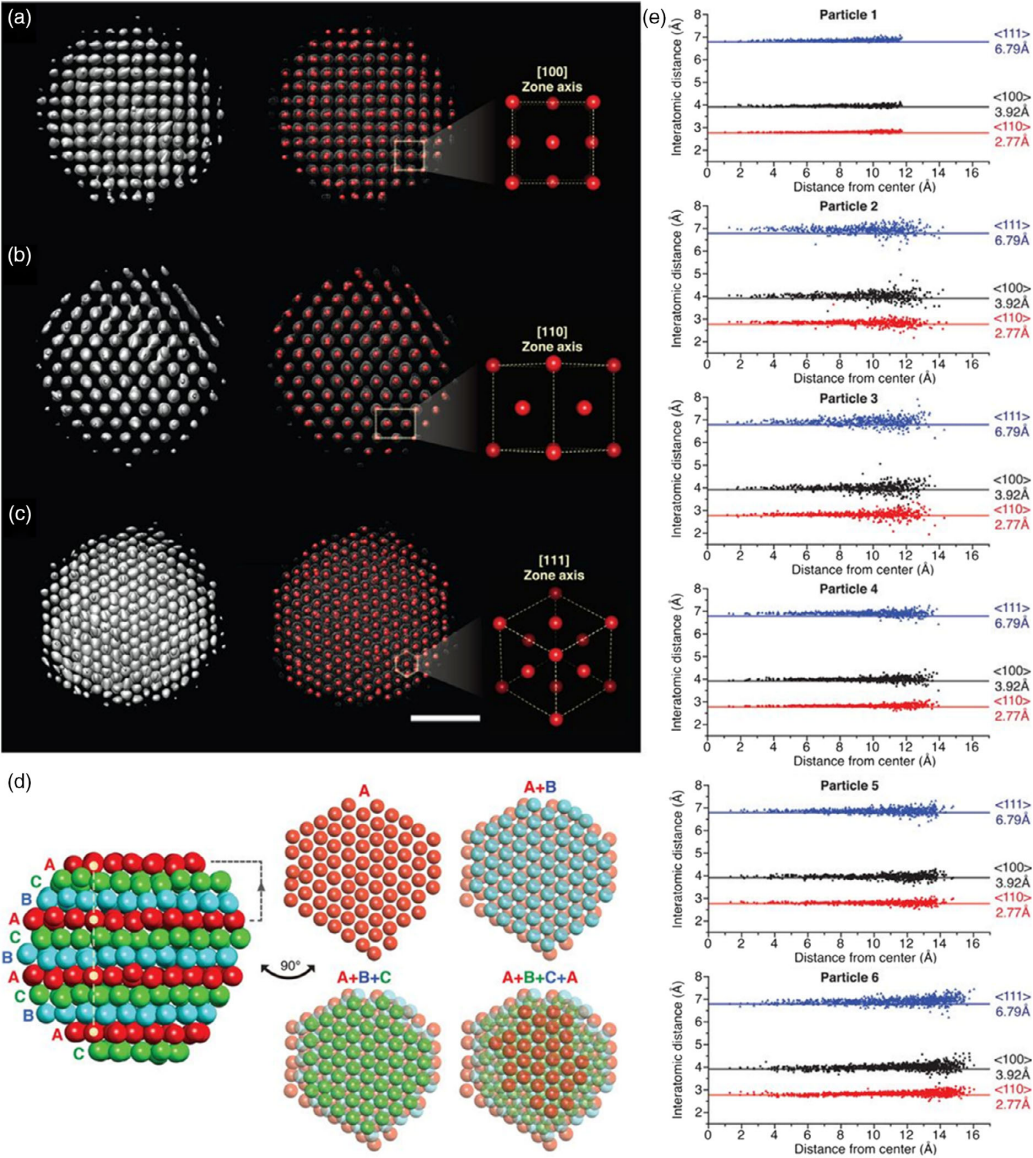
a–c) 3D density maps and corresponding atomic maps of 2.5 nm‐sized platinum nanoparticles reconstructed by Brownian one particle reconstruction with [100], [110], and [111] zone axes. d) ABC stacking of the 3D map indicating an fcc structure. e) Interatomic distances of six reconstructed nanoparticles showing deviation near the surfaces. Scale bar, 1 nm. Reproduced with permission.^[^ ^27^ ^]^ Copyright 2020, American Association for the Advancement of Science.

The 3D structures of platinum nanoparticles were successfully analyzed from 3D reconstructed maps with atomic‐level resolution. **Figure** **5**a–c shows a representative 2.52 nm platinum nanoparticle. Platinum nanoparticles share a typical single crystalline fcc structure, as revealed by the atomic arrangements in the <100>, <110>, and <111> zone axes. The hexagonal close‐packed arrays of atoms on the (111) plane are confirmed (Figure 5c). The fcc structure can also be determined by the packing sequence of the hexagonal (111) array, with ABC stacking (Figure 5d).

It has long been thought that nanoparticles are not perfect crystals due to point defects inside the crystal and lattice expansion or compression near their surfaces.^[^ ^16^, ^28^, ^66^ ^]^ The interatomic distances between constituent atoms are plotted according to the radial distance from the center of a nanoparticle to investigate the degree of variation in crystallinity. While the atomic distances are almost constant near the core, they fluctuate slightly near the surface (Figure 5e). The observed lattice parameter deviation originates from the lack of ordering of surface atoms, which are influenced by high surface energy. The lattice parameters of 2.4, 2.5, and 2.7 nm‐sized platinum nanoparticles are fitted to 4.02, 3.99, and 3.96 Å, respectively, which are 2.56%, 1.74%, and 0.95% larger than the bulk lattice parameter. Notably, this tendency is opposite that of the lattice parameters of vacuum‐exposed nanoparticles.^[^ ^30^ ^]^ The lattice parameters of metal nanoparticles with bare surfaces and without surface ligands and solvent interactions are generally compressed due to the effect of surface tension and low coordination numbers.^[^ ^36^, ^67^ ^]^ Density functional theory (DFT) calculation was used to confirm that the binding of poly(vinylpyrrolidone) (PVP) ligands in solution phase weakens the metallic bonding between platinum atoms near the surface, resulting in a slight lattice expansion of colloidal platinum nanoparticles.

A strain analysis of a single particle is important for understanding the catalytic properties of metal nanoparticles, because local strain determines the catalytic activity of the nanoparticles by regulating the adsorption and desorption of reactants.^[^ ^68^ ^]^ Platinum nanoparticles with tensile strain exhibit high efficiency of the methanol oxidation reaction,^[^ ^69^ ^]^ whereas compressed platinum particles enhance the oxygen reduction reaction.^[^ ^70^ ^]^ The high catalytic effect of strained nanoparticles emphasizes the importance of strain analysis. Currently, strain can be characterized using 2D TEM image or X‐ray diffraction techniques, but these methods cannot provide strain tensors applied on each atom.^[^ ^71^ ^]^

**Figure 6:**
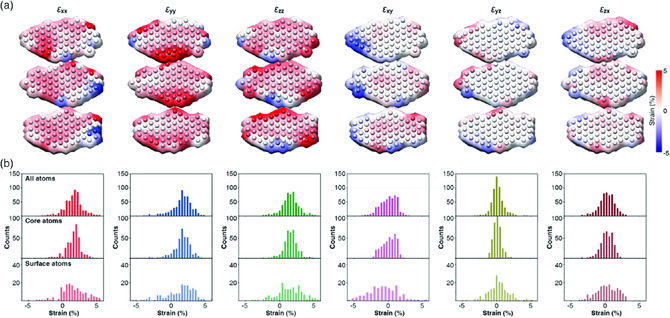
a) Strain maps of three consecutive atomic layers with six strain tensor components of a 2.5 nm‐sized platinum nanoparticle. b) Strain distribution of (top) all constituent atoms, (middle) atoms at the core, and (bottom) atoms near the surface of the nanoparticle. Reproduced with permission.^[^ ^27^ ^]^ Copyright 2020, American Association for the Advancement of Science.

Using the perfect fcc as a reference, a full set of 3D strain maps was obtained (**Figure** **6**). The strain maps of the 2.5 nm nanoparticle clearly reveal large tensile strains on the surface atoms compared with the core atoms (Figure 6). The strain distributions of the surface atoms are much broader than those of the core atoms, indicating that the surface atoms are more disordered. Tensile strains are found throughout a nanoparticle, from surface to core, due to lattice expansion.

Brownian one particle reconstruction has several advantages over the 3D electron tomography method. While 3D electron tomography is conducted under an ultra‐high vacuum, which can induce deformation of a nanoparticle from its native state, Brownian one particle reconstruction is conducted in a liquid; this is useful, because nanoparticles are often used in liquid media. The projection orientations of the TEM images for Brownian one particle reconstruction permit coverage of the full range of angles without the missing wedge problem. In addition, concurrently tracking many small nanoparticles in liquid within one field of view is possible, allowing for parallel reconstruction of multiple heterogeneous nanoparticles. However, it is necessary to control the chemical conditions during a prolonged TEM imaging session in a liquid cell, because the electron beam might perturb the solvent molecules. Solvent should be carefully chosen to avoid sample degradation by reaction between solvent molecule and electron beam, which can be aided by quantitative analysis of chemical effect of electron beam.^[^ ^72^ ^]^ Currently, a size of nanoparticles analyzed by Brownian one particle reconstruction is in a range of less than 5 nm, and it requires further research for larger sized nanoparticles.^[^ ^27^, ^64^ ^]^

## 4 Perspectives

X‐ray crystallography, which can be used to characterize the 3D structures of bulk materials and the average structures of nanomaterials, has dominated materials science in the last century. Since the 1990s, TEM has been considered an essential imaging modality for characterizing the crystal structures of individual nanomaterials. The 3D electron tomography and Brownian one particle reconstruction were developed as a means of visualizing the 3D structures of individual nanoparticles. Recent advances in TEM resolution, reconstruction algorithms, and liquid‐phase TEM have greatly improved the spatial resolution of these innovative characterization methodologies. The methods are now blossoming as they allow investigation of the structures and properties of new materials based on 3D maps. Currently, 3D atomic coordinates are mainly acquired for nanoparticles composed of a single heavy metal element, such as gold nanoparticles or platinum nanoparticles. Thus, future research will focus on technical developments that make it possible to obtain the 3D atomic structures of nanoparticles with light elements.

It is currently challenging to decipher the atomic structures of multi‐component nanoparticles, which are important for catalysts and optics. Yang et al. reported on classification of the individual elements of alloy particles from a 3D density map reconstructed via 3D electron tomography.^[^ ^73^, ^74^ ^]^ The authors obtained the 3D atomic coordinates of an 8.4 nm‐sized Fe_0.28_Pt_0.72_ nanoparticle. All 23 196 potential atomic positions were assigned to 6569 iron atoms and 16 627 platinum atoms on the basis of intensity variation, because STEM intensity is highly dependent on atomic weight.^[^ ^73^ ^]^ As the atomic weights of the Pt (*Z* = 78) and Fe (*Z* = 26) atoms in FePt nanoparticles differ by a factor of 3, Pt and Fe atoms can be identified using the difference in intensity in the 3D density map. The classification methodology needs to be improved, such that the atomic structures of important alloy particles with smaller atomic weight differences, such as Ag/Au nanoparticles, can be resolved.^[^ ^75^, ^76^ ^]^ Further development of 3D reconstruction methodologies will allow identification of the atomic structures of various types of materials, such as metal oxides, sulfides, selenides, and phosphides, and more complex materials, such as core–shell structures, doping materials, Janus structures, and alloy materials.

Determination of the 3D atomic positions of amorphous materials is another important and challenging goal of 3D reconstruction methodologies.^[^ ^77^ ^]^ Amorphous materials are very common and important materials, but their detailed structures have not been defined, because conventional X‐ray scattering‐based methodologies are not applicable to amorphous materials. The use of 3D reconstruction methodologies to assign the atomic positions of amorphous materials has been demonstrated. Simulated TEM images of model amorphous glass particles with 55 different projection orientations were 3D reconstructed. The reconstructed atomic structure was confirmed to be identical to the original model structure. The key to reconstruct the 3D atomic coordinates of amorphous particles is obtaining high‐resolution TEM images with high contrast and reduced blurring. Similarly, visualization of quasicrystals is another goal of 3D reconstruction methodologies.

The ultimate goal of the structural study of materials is to understand their chemical and physical properties based on the structure. To fundamentally understand the correlation between structure and property, the 3D structural analysis must be repeatable and reproducible for a statistically large number of nanoparticles. The recent reports of 3D structural analysis based on TEM considered the atomic structures of only a few nanoparticles. Improving the data acquisition rate, data processing speed, and speed of 3D reconstruction are key advancements that await intensive efforts. Compensating the limitations of one technique by combining two or more techniques other techniques, such as nanobeam electron diffraction, can be another breakthrough for solving such problems.^[^ ^78^ ^]^

We believe that the structural determination of individual nanoparticles along with the further development of 3D reconstruction methodologies, including 3D electron tomography and Brownian one particle reconstruction, will transform not only materials science but also computational chemistry. Currently, calculation‐based studies of nanoparticles are based on theoretically estimated crystal structures. Thus, the accurate 3D atomic positions of each nanoparticle obtained from 3D structural analysis can be directly applied to theoretical calculations, which is likely to improve the accuracy of those calculations. Accurate 3D reconstruction‐based calculations will aid in the design of functional nanomaterials with desired chemical activities.

## Conflict of Interest

The authors declare no conflict of interest.
